# Psychosocial factors associated with engagement and efficacy of mindfulness intervention in the classroom

**DOI:** 10.1371/journal.pone.0352195

**Published:** 2026-06-25

**Authors:** Savannah K.H. Siew, Junhong Yu

**Affiliations:** 1 Psychology, School of Social Sciences, Nanyang Technological University, Singapore, Singapore; 2 Yeo Boon Khim Mind Science Centre, National University of Singapore, Singapore, Singapore; Dong-A University College of Business Administration, KOREA, REPUBLIC OF

## Abstract

**Background:**

Mindfulness practice has been associated with a range of benefits in the literature. However, intervention effects show substantial person-to-person variability, and the individual differences underlying this variability remain underexamined. Identifying the psychosocial predictors of the practice can increase our understanding of the mechanisms underlying the benefits of mindfulness practice and inform strategies to increase both engagement and efficacy of it.

**Methodology:**

66 university students in Singapore were recruited if they had completed a course where mindfulness practice was conducted during the lecture. Participants were aged between 18–39 years old (Mage = 22.2 years). They completed an online survey measuring trait mindfulness, personality traits, attachment and conflict styles, intrinsic motivation, grit, and attention control. Outcome variables of engagement and efficacy were self-reported. Multiple linear regression for each independent variable controlled for different course taken and prior mindfulness experience using a multiple comparison corrected confidence interval of 99.8%.

**Results:**

Intrinsic motivation was positively associated with both engagement in mindfulness practice and its usefulness. The observing facet of trait mindfulness, total trait mindfulness and conscientious personality were negatively associated with how difficult participants felt the mindfulness practice was.

**Discussion:**

The significant psychosocial and cognitive variables associated with both engagement and efficacy of mindfulness practice from this exploratory study add to our understanding of the type of participants that might be more suited for mindfulness practice in the classroom.

## Introduction

Mindfulness is a concept characterized by present-moment awareness, coupled with a stance of non-judgement towards that awareness [1]. It can be practiced through a range of guided and non-guided forms, including breath awareness, loving-kindness meditation, and mindfulness integrated into daily activities. Mindfulness practice has been associated with benefits across physical [2,3], mental [3–6], cognitive [3,4,7,8] and social health outcomes [9,10] in healthy populations, and in the classroom setting specifically, has been shown to improve learning [11–13] and reduce stress [14,15]. However, intervention effects show substantial person-to-person variability. Generally, most researchers believe that mindfulness can be partially evoked by situational and environmental factors although some inherent qualities result in individual differences regarding the practice [16,17].

Emerging research evidence for individual differences in mindfulness intervention reveals some potential factors. In meta-analyses on mindfulness interventions for depression in both older and emerging adults, Asians showed greater improvements compared to Europeans and North Americans which the authors postulated might be due to familiarity as mindfulness has its roots in Eastern teachings [18,19]. Among diabetic patients with comorbid anxiety and depression symptoms, men and those higher in the personality trait of extraversion benefitted less from MBCT [20]. Other personality factors seem to moderate the effects of mindfulness practice on levels of burnout for professionals working in mental health. Participants who were lower in vigilance and emotional stability had better outcomes following the mindfulness intervention [21]. These personality factors correlated with higher agreeableness and neuroticism respectively in terms of the big five personality traits.

Additionally, lower nonreactivity, a trait mindfulness facet, at baseline was associated with greater improvements in mindfulness meditation while higher nonreactivity at baseline was associated with greater improvements in Mindfulness-based Cognitive Therapy (MBCT) [22]. MBCT is a type of psychotherapy that combines Cognitive Behavioral Therapy with mindfulness meditation. In young adults with cancer, factors significantly associated with greater improvements in anxiety symptoms after mindfulness-based intervention include higher physical functioning at baseline, higher anxiety and fatigue scores, sleep disturbances and those who were female [23]. Overall, it seems that individual characteristics such as race, sex, personality traits and the mindfulness aspect of non-reactivity could account for differences in mindfulness efficacy. Across these studies, participants are drawn predominantly from specific clinical populations. Moreover, there are still limited variables that have been studied and more diverse research is necessary to fully understand individual differences in mindfulness interventions.

Some of these individual difference variables that are associated with mindfulness may predict efficacy and engagement in the practice but have not been studied in this context. Trait mindfulness and attachment styles are associated, but this relationship tends to be only in the forward direction, looking at how the former affects the latter [24]. An anxious attachment was found to mediate the association between mindfulness and psychological distress [25]. However, these cross-sectional studies are not able to distinguish the direction of the association and it is possible that attachment styles could also affect one’s ability to engage in mindfulness practice. Internal working models developed from early attachment experiences may shape self-regulatory ability, which in turn may influence engagement with mindfulness. The interpersonal conflict style of control is also associated with dispositional mindfulness [26], which is reasonable given that it is at odds with characterizations of what mindfulness entails. Moreover, mindfulness requires practice and given that it is often something that most people do not do regularly, engaging with it may require intrinsic motivation, grit or attention control. Mindfulness and grit have been found to be associated [27] and grit predicted mindfulness levels in student-athletes [28].

Following mindfulness interventions, there is substantial person-to-person variability in outcomes [29]. Hence, there is a need to examine individual differences. Within the mindfulness research sphere, the gap in the current literature points to a lack of studies looking at individual characteristics predicting engagement during the mindfulness practice, despite general consensus that both situational and inherent factors contribute to individual differences in mindfulness. Most studies tend to focus on situational factors, investigating the effects of different types of practice, as well as the duration and frequency of practice, on mindfulness engagement and its benefits. However, inherent individual characteristics are less studied.

Engagement refers to their experience of the mindfulness practice and in particular, focuses on the level of engagement during the practice and how they perceive the difficulty level of the practice to be. Previous studies tend to focus on the moderating effect of individual differences on mindfulness practice efficacy, which refers to the outcome and benefits. With mindfulness being a practice requiring active involvement, engagement levels could be an important factor to examine to understand its impact. Even so, studies looking into individual differences factors are still nascent, as previously mentioned, and there are several other variables that show an association with the variable of mindfulness that have not been included in such research. Early findings identify several predictors of mindfulness efficacy but more research is needed to identify additional relevant variables.

Furthermore, most of these studies looked at specific demographics, with majority being clinical populations. Young adults could be a particularly good target to study since they are at a suitable age where they can understand the purpose and meaning of mindfulness practice and are potentially able to develop an interest in a practice that could be beneficial in their daily lives from then on. Moreover, when examining the beliefs among Western college young adults, most viewed mindfulness as a secular practice that can be accessed and has benefits [30]. Additionally, mindfulness has been found to positively influence cognitive outcomes [31,32] and academic performances [33,34]. To the best of our knowledge, no current studies looked at individual differences that influence the effects of mindfulness interventions in the classroom.

The selected individual difference variables in this study span three broad conceptual domains relevant to mindfulness engagement and efficacy. The first comprises trait-level constructs closely related to mindfulness itself, including dispositional mindfulness and attention control, which capture an individual’s baseline tendency and capacity for present-moment awareness. The second includes broader personality and interpersonal factors, namely personality traits, attachment, and conflict styles, which shape how individuals approach novel practices and regulate themselves under emotional or interpersonal challenge. The third involves motivational and self-regulatory factors, namely intrinsic motivation and grit, which reflect the likelihood of engaging with and persisting in the practice. Across these domains, engagement and perceived efficacy of mindfulness practice are expected to be higher among individuals with greater baseline capacities for awareness and attention, more constructive interpersonal tendencies, and stronger motivational orientations.

### Overview of the study

Although individual differences in mindfulness outcomes have been documented across specific clinical populations and demographic factors, much less is known about how psychosocial and cognitive variables relate to engagement and perceived efficacy of mindfulness in non-clinical university populations. The present study addresses this gap by examining a broad set of predictors of engagement and efficacy of mindfulness practice in university students who completed a classroom mindfulness intervention. The predictors studied include trait mindfulness, personality traits, attachment and conflict styles, intrinsic motivation, grit, and attention control, with engagement and efficacy of the mindfulness practice self-reported as outcomes. As this is an exploratory study, it is hypothesised that some of these predictors will show significant associations with the outcome variables. Separate multiple linear regressions were conducted for each predictor, controlling for course taken and prior mindfulness experience, with significance assessed using a multiple comparison corrected confidence interval of 99.8%.

## Materials and methods

### Participants and procedures

Participants were university students recruited from Nanyang Technological University between 3 April 2023–16 December 2023. Mass recruitment emails were sent to all students by the social science undergraduate office and specific announcements were additionally sent out to courses that had the mindfulness practice implemented during this period. The inclusion criteria for the study were to have completed one course that had a mindfulness practice at the start of every lecture and attended the mindfulness sessions at least 70% of the time, since it was not compulsory.

Having mindfulness practices before the start of lectures was not a common practice of the university nor the country. During the time of the study, there were two courses that implemented such mindfulness practice. In the first, participants had a 10-minute guided mindfulness session before the start of every lecture for 12 weeks. For the other course, participants were guided through a mindfulness session online during the class for 6 weeks. Mindfulness sessions included breath awareness, mindful perception, and loving-kindness sessions and were guided by the instructor.

Interested participants were sent the online survey, provided written informed consent, and received SGD$5 remuneration upon completion. Some students were minors and in these cases, written informed consent was also obtained from their parents/guardians. Ethical approval was granted by the Nanyang Technological University’s Institutional Review Board (Ref No. IRB-2023–073). A priori calculations suggest that a sample size of 55 is adequate to detect medium effects (i.e., *f*^2^ = 0.15), according to effect sizes of past related studies, with a power of 0.8 for one tested predictor and three total predictors. 66 participants completed the questionnaires used in this study with detailed descriptions of all the measures expanded on in the next section. Table 1 shows the sociodemographic information of the participants.

**Table 1:** Sociodemographic Information of Participants.

	Mean (S.D.)/Frequency (%)
Age (n = 64)	22.17 (3.95)
Sex	
Female	37 (56.1%)
Male	29 (43.9%)
Housing (n = 62)	
1-3 room HDB	10 (16.1%)
4-5 room/executive HDB	30 (48.4%)
Private Housing	22 (35.5%)
Religion (n = 64)	
Christian/Catholic	14 (21.9%)
Buddhist/Taoist	22 (34.4%)
Hindu	7 (10.9%)
Islam	4 (6.3%)
None	17 (26.6%)
Current year of education	
Year 1 undergraduate	37 (56.1%)
Year 2 undergraduate	10 (15.2%)
Year 3 undergraduate	9 (13.6%)
Year 4 undergraduate	3 (4.5%)
Post-graduate/Graduated	7 (10.6%)
Course of study (n = 65)	
Psychology and related studies	17 (26.2%)
Engineering	18 (27.7%)
Humanities (Philosophy, Linguistics, Sociology)	6 (9.2%)
Economics, Science, Business, Computing	24 (36.9%)
Prior mindfulness experience (n = 65)	
Yes	27 (41.5%)
No	38 (58.5%)

Note. N = 66 unless otherwise specified due to missing data. HDB = Housing development board, which are government-subsidized housing.

### Measures

The survey included demographic questions on age, sex, religion, current year of education, socioeconomic status, course of study, and prior mindfulness experience outside of the classroom session. Socioeconomic status is represented by housing type. In Singapore, housing is the largest expenditure of one’s lifetime given the exceptionally high prices. Thus, housing type is a sensitive indicator of SES in Singapore [35]. The following predictive variables were included in the online survey.

#### Trait mindfulness.

The 15-item Five-Facet Mindfulness Questionnaire (FFMQ-15) [36] was used to measure trait mindfulness. This questionnaire is preferable to the Mindful Attention Awareness Scale [37] which focused only on one dimension of mindfulness, acting with awareness. The FFMQ is more comprehensive and assesses five facets: (1) Acting with Awareness, (2) Observing, (3) Describing, (4) Non-judgement, and (5) Non-reactivity. Acting with awareness involves attention to the present moment, observing refers to taking note of experiences both internally and from the external environment, while describing is the ability to express experiences with words. Non-judgement includes being accepting of one’s inner experiences such as our thoughts and emotions without assigning morality to them, while non-reactivity is the ability to disengage from our inner experiences, letting them occur without getting attached to them.

The FFMQ was rated on a five-point Likert scale, with higher scores representing higher agreement or occurrence of these mindfulness facets. A total trait mindfulness score was also computed which is the sum of all facets scores. The FFMQ-15 has been established as a valid alternative to the original 39-item questionnaire in a mindfulness intervention study [38]. Additionally, its validity among a similar demographic of university students has been established [39]. The FFMQ-15 had an acceptable internal consistency reliability estimate (ω = 0.74).

#### Personality traits.

This was measured by the 30-item NEO Five-Factor Inventory questionnaire which was validated with a student sample from six countries from varying regions and established to have comparable reliability and validity to the longer versions [40]. There were six questions for each big five personality trait: (1) Openness to experience, (2) Conscientiousness, (3) Extraversion, (4) Agreeableness, and (5) Neuroticism. Each statement had a description and participants had to rate how strongly they agree that the statement describes themselves on a seven-point Likert scale.

Scores for each personality scale were summed and a higher score represented a stronger relation to that personality trait. Each of the personality subscales had acceptable to good internal consistency: openness (ω = 0.75), conscientiousness (ω = 0.65), extraversion (ω = 0.86), agreeableness (ω = 0.67), and neuroticism (ω = 0.77).

#### Attachment style.

The 15-item Attachment Style Questionnaire – Short Form (ASQ-SF) [41] was used to measure attachment styles and was developed specifically for the Asian cultural context. This is particularly important because key cultural differences between parent-child relationships and attitudes and behaviors toward attachment and relationships will affect the cross-cultural validity of previously developed questionnaires for use in non-western cultures. The same four attachment styles of secure, fearful-avoidant, preoccupied, and dismissive attachments were present in the Asian sample.

On a five-point Likert scale, participants chose how much they agree with the statements presented and scores for all four styles of attachment were calculated. This is a more appropriate questionnaire than the widely popular Adult Attachment Scale which focuses on romantic or intimate relationships that some in our sample might not have encountered yet. Scales for the ASQ-SF showed acceptable to good internal consistency: fearful-avoidant (ω = 0.88), preoccupied (ω = 0.89), and dismissive (ω = 0.70), except for secure attachment (ω = 0.35). The poor reliability estimate could reflect the fewer items on this subscale, particularly in the short form version.

#### Interpersonal conflict styles.

This was measured using Rahim’s Organizational Conflict Inventory – II (ROC II) [42]. Form C, which focuses on how conflicts with peers are handled, was the most suitable for our participants. The five types of conflict styles are (1) collaborating, (2) accommodating, (3) competing, (4) avoiding, and (5) compromising. Each item was scored on a 5-point Likert scale with a higher score representing greater use of the conflict style. The inventory has good reliability and validity and most of the scales were shown to be subjected to low social desirability bias. Most subscales on the ROC II showed excellent internal consistency: collaborating (ω = 0.88), accommodating (ω = 0.87), competing (ω = 0.86), avoiding (ω = 0.90), except for compromising (ω = 0.65), which had fewer items than the other subscales.

#### Intrinsic motivation.

The 7-item interest/enjoyment subscale of the Intrinsic Motivation Inventory (IMI) [43] was used to measure motivation as it is considered a self-report of intrinsic motivation. On a 7-point Likert Scale, participants rated how true each statement was for them with regards to the mindfulness activity and a higher score represented higher intrinsic motivation. The IMI had excellent internal consistency with a reliability estimate coefficient of 0.95.

#### Grit.

This was measured using the 8-item Short Grit Scale (Grit-S) [44] and has been validated to be psychometrically superior to the original scale across a range of age groups. The self-report measure is scored on a 5-point Likert scale and higher scores on the scale represent higher grit. The Grit-S had good internal consistency with a reliability estimate coefficient of 0.80.

#### Attention.

The 20-item Attention Control Scale (ACS) [45] was used as a self-report measurement of the cognitive variable of attention, more specifically, the ability to voluntarily control one’s attention. Since this was an online survey, administering cognitive tasks was not feasible in this online format, and this measure was the most suitable alternative. It assesses two different constructs of attention, namely attention shifting and attention focusing. The items are scored on a 4-point Likert scale with higher scores representing better attention control. The ACS had a good internal consistency reliability estimate (ω = 0.83).

#### Outcome variables.

**Fig 1:**
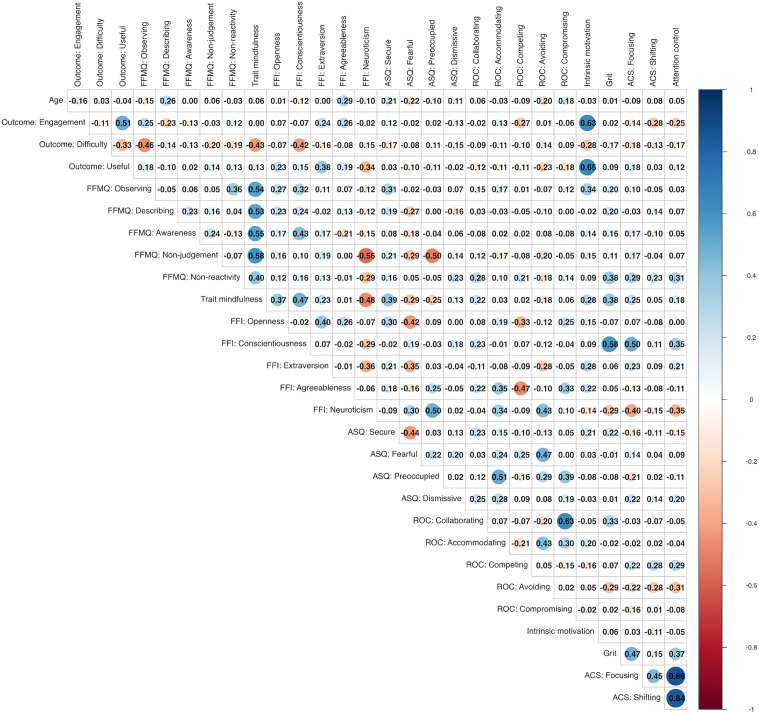
Correlation Heat Map of All Variables. The numbers indicate Pearson’s correlation coefficient. Color circles corresponding with the magnitude and direction of the correlation coefficient are only shown for significant pairs at p < .05. Among the strongest correlations with the outcome variables, intrinsic motivation showed positive associations with both engagement and perceived usefulness. Perceived difficulty was negatively correlated with the observing facet of trait mindfulness, total trait mindfulness, and conscientiousness. The trait mindfulness facets clustered together with moderate to strong inter-correlations, and attention control subscales showing very strong inter-correlations. FFMQ = Five-facet mindfulness questionnaire, FFI = Five factor inventory of personality traits, ASQ = Attachment style questionnaire, ROC = Rahim’s organizational conflict inventory, ACS = Attention control scale.

Engagement and efficacy of the brief mindfulness practice in class were measured using a self-developed questionnaire on a 5-point Likert scale. The three questions included how engaged they were during the practice, how difficult the mindfulness session was, and how useful they think the practice is for them. The three outcome variables were treated as an ordinal approximation of a continuous variable [46,47]. Self-developed questions were used because there are no validated instruments for assessing engagement with a mindfulness intervention at the current moment. However, the construct validity of these measures can be observed by a significant large positive correlation between engagement and usefulness, and a significant medium negative correlation between difficulty and usefulness (see Fig 1.). Moreover, both engagement and difficulty were correlated with trait mindfulness measures in the direction that one would expect.

### Statistical analysis

All analyses were performed in R 4.0.3. Descriptive statistical analysis was carried out by using Pearson’s correlation to explore the relationships between the continuous independent and outcome variables. Chi-square test was used for categorical independent variables. Statistical significance was set at *p* < .05. Multiple linear regression was carried out to test for significant relationships between each predictor and outcome variables while controlling for the different course taken and prior mindfulness experience. Multicollinearity between the predictive variable and control variables was checked by ensuring the Variance Inflation Factor value does not exceed 5. To correct for multiple comparisons, a corrected confidence interval of 99.8% was used as 25 predictors were analysed. All data and R code for the analysis used in this study have been made available on https://osf.io/mkvc8/?view_only=0d0ffdfdd5454a66906586dc4e3c8a64.

## Results

The Pearson’s correlation coefficients between all continuous variables used in this study are shown in Fig 1. All categorical independent variables, which included sex, religion, current year of education, housing type, used as a representation of socioeconomic status, course of study and prior mindfulness experience outside of the classroom session were not significantly correlated with the outcome variables.

**Fig 2:**
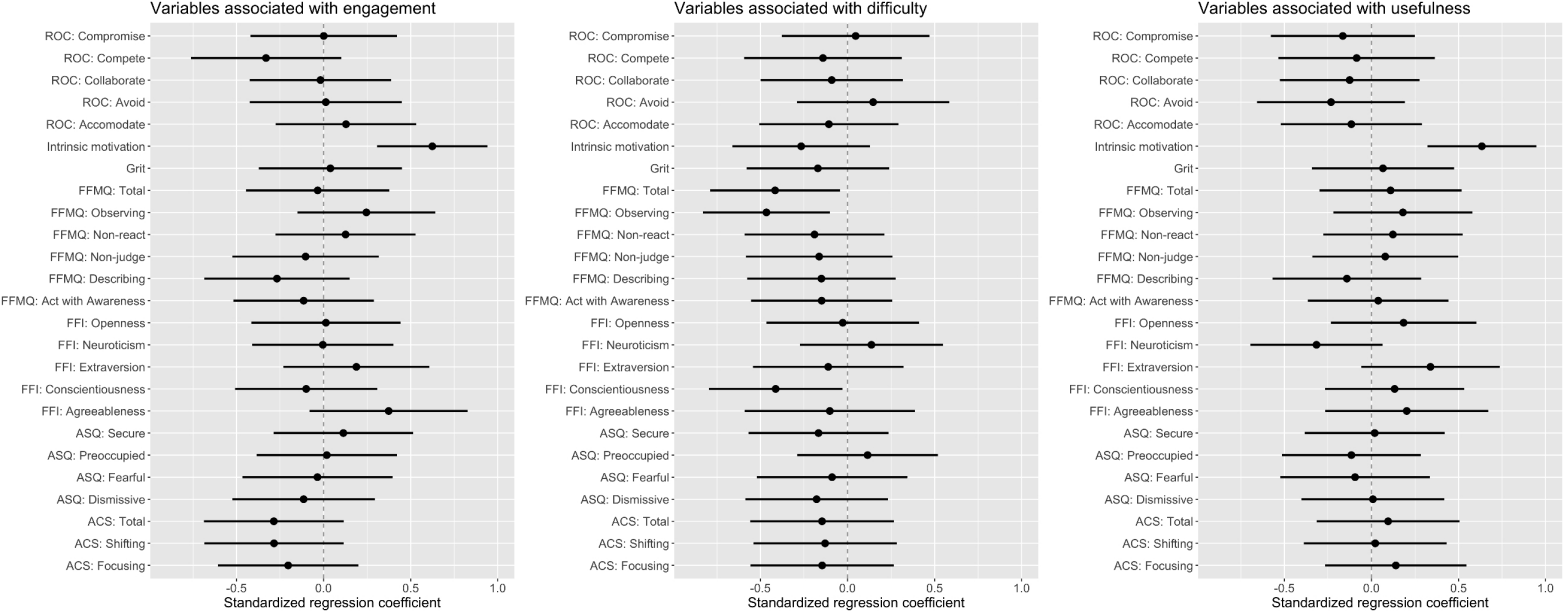
Forest Plot of Standardized Regression Coefficients for All Predictor Variables on the Three Mindfulness Practice Outcomes. Standardized regression coefficients are represented by dots within their respective lines that correspond to their 99.8% confidence intervals. Predictors whose confidence intervals do not cross zero were considered significant. For engagement, intrinsic motivation emerged as the only significant positive predictor. For perceived difficulty, the observing facet of trait mindfulness, total trait mindfulness, and conscientiousness emerged as significant negative predictors, indicating that participants higher on these traits reported finding the practice less difficult. For perceived usefulness, intrinsic motivation again emerged as a significant positive predictor. Other predictor variables, including attachment styles, conflict styles, grit, and attention control, did not show significant associations with any outcome after multiple comparison correction. ROC = Rahim’s organizational conflict inventory, FFMQ = Five-facet mindfulness questionnaire, FFI = Five factor inventory of personality traits, ASQ = Attachment style questionnaire, ACS = Attention control scale.

The results of the multiple linear regressions for each predictor were shown for all three outcome variables in Fig 2, with all significant predictors showing a moderate to large effect size. Intrinsic motivation significantly predicted both engagement with mindfulness practice (β = 0.62, *p* < .001*;R*^*2*^ = 0.44, *F*(3, 60) = 15.91, *p* < .001) and its usefulness (β = 0.63, *p* < .001*;R*^*2*^ = 0.44, *F*(3, 60) = 15.91, *p* < .001). Higher conscientious personality (β = −0.41, *p* = .001*;R*^*2*^ = 0.21, *F*(3, 59) = 5.09, *p* = .003), observing facet of trait mindfulness (β = −0.47, *p* < .001*;R*^*2*^ = 0.25, *F*(3, 61) = 6.85, *p* < .001) and total trait mindfulness (β = −0.42, *p* = .001*;R*^*2*^ = 0.21, *F*(3, 60) = 5.16, *p* = .003) significantly predicted lower difficulty of mindfulness practice.

## Discussion

Our study aimed to uncover the psychosocial and cognitive variables significantly associated with both engagement and efficacy of mindfulness practice. Generally, these individual difference factors included trait mindfulness, personality traits and intrinsic motivation after controlling for the two courses that participants attended and prior mindfulness experience.

The observing facet of mindfulness refers to taking note of experiences both internally and from the external environment. Those higher in this trait reported finding the mindfulness practice less difficult. Especially during one’s initial exposure to the practice, most of the focus is on the awareness aspect of mindfulness and thus, these individuals are already better at that skill and are likely to find the practice less difficult. This also lends support to the common analogy of mindfulness as a muscle that has to be repeatedly practiced to be strengthened. This analogy is further supported by evidence of neuroplasticity following mindfulness interventions where brain structures relating to sustained attention show volumetric increases [48]. This suggests a self-reinforcing cycle where practicing mindfulness can lead to higher observation of both internal and external experiences [49] which in turn makes subsequent practice less difficult. This mechanism possibly explains findings of the frequency of mindfulness practice correlating with better outcomes [50].

The other facets of trait mindfulness did not show a significant association with either engagement or outcomes. This is surprising given that a previous network analysis found that Acting with Awareness has a key role to play in being the driving force of mindfulness due to its high interconnectedness with the other facets and centrality estimate [51]. However, it could be that the observing facet may map most directly onto the simple act of attentional observation, while the others possibly rely on higher-order actions, and are thus significantly related to engagement.

Our findings regarding personality traits reveal slightly inconsistent results with mindfulness studies looking at the five personality traits. Trait mindfulness has been found to be most consistently associated with neuroticism and conscientiousness [52], and has smaller associations with other personality traits [53]. The most recent meta-analysis revealed trait mindfulness to be significantly associated with all five personality traits, with the strongest association for neuroticism, followed by conscientiousness [54]. However, when looking at predictors of mindfulness intervention outcomes and engagement, only some personality traits are significant. In particular, those higher in neuroticism [21,55] and agreeableness [21], and lower in extraversion [20] showed stronger intervention effects. The personality traits of conscientiousness and openness were associated with adherence to a mindfulness intervention [56].

In our study, we only found that those who were more conscientious found the practice less difficult. The personality trait of conscientiousness refers to those who are more self-disciplined and goal-directed, which seems to reflect greater self-awareness when engaging with mindfulness practice. It is important to note that some of these differences in personality findings could be due to differences in participants from healthy versus clinical populations and the different outcome measures explored. Moreover, it seems that all the personality traits may be associated with trait mindfulness, but only some personality traits predict mindfulness intervention outcomes.

Intrinsic motivation proves to be an important factor in predicting both engagement and efficacy, showing a large effect size in relation to both outcome variables. Intrinsic motivation refers to doing a task for internal satisfaction rather than external rewards. This factor may predict engagement and efficacy in mindfulness because someone who is intrinsically motivated may be more likely to sustain the practice. Self-Determination Theory explains how intrinsic motivation drives engagement and positive behaviors because it fulfils our psychological need for autonomy and competence [57]. Mindfulness stands in contrast to the constant distraction characterizing contemporary daily life, largely perpetuated by widespread technological access to social connection and information. Those who are more externally motivated may find it harder to engage with the practice given greater temptations outside that provide instant gratification. Intrinsically motivated individuals may be better able to disregard such distractions and sustain practice despite initial difficulty. Research into the association between trait mindfulness and intrinsic motivation shows a significant relationship [58]. However, intrinsic motivation is often explored as an outcome variable in mindfulness intervention studies instead of a potential moderator [58].

Other factors studied here, namely conflict styles, attachment styles, grit, and attention control, did not show significant associations with engagement and efficacy of mindfulness practice. This means that individual differences in these factors should not affect the outcomes and engagement with the practice. Previous studies have looked at differences between meditation-naïve participants and those with meditation experience [59]. Therefore, it was important to control for prior mindfulness experience in our current sample to find predictors that were relevant to both groups, regardless of how far along they are in their journey with mindfulness practice. Moreover, there has been plenty of research demonstrating the importance of repeated practice during mindfulness for the cultivation of mindful awareness. The impact of the individual difference variables uncovered in this study holds true regardless of whether the participant is meditation-naïve or had prior experience. This also highlights to researchers the importance of considering individual differences in future mindfulness research since it could moderate the findings and provide insight into other factors that could possibly predict mindfulness engagement and benefits.

The significance of the results presented needs to be considered within the context of a few limitations. First, it is important to note that this is a cross-sectional study which impedes our ability to make any directional assumptions about the relationship found. However, this exploratory study has helped to narrow down to a few important variables that require further longitudinal investigation into how they affect individual differences in both engagement and efficacy of mindfulness practice. Relatedly, some potential confounding variables that could have influenced the relationship were not included such as academic or personal stress levels.

Second, while each model’s predictor-to-sample-size ratio falls within conventional bounds because each main predictor was tested in a separate regression with two covariates, giving a ratio of 1:22, the overall sample size remains modest. Future research could extend the present exploratory analyses by testing partial models that group predictors within conceptual domains, allowing assessment of incremental contributions within each domain. A priori confirmatory tests of the most promising predictors identified here can then be analysed with adequately powered replications.

Third, the current exploratory study looks at participants’ engagement and efficacy of their mindfulness practice in naturalistic settings according to their self-report results which could increase common method bias. On one hand, it provides valuable data points since perception of their own engagement and efficacy in undergoing the mindfulness practice is of high importance. On the other hand, more specific measurements, such as trait mindfulness scores, and objective measurements, like brain activity from functional magnetic resonance imaging scans during mindfulness-related tasks, could provide another significant perspective to the picture. Moreover, most of the established questionnaires in the study have put in place strategies to mitigate common risk bias by including reverse-scored questions.

Fourth, the current study was conducted within a specific cultural and educational context, which may limit the external validity of findings to other cultural settings. We sought to collect data on religion, as mindfulness is more closely linked to some than others, and had a relatively diverse sample in terms of religious makeup. Thus, the cultural context of this sample might not significantly impact the external validity of the results to cross-cultural samples. However, this needs to be verified by conducting cross-cultural comparative studies or by replicating this study in a different population. Last, our findings of individual differences within mindfulness practice may not extend to all types of mindfulness practice. Only awareness of breath, body awareness, mindful perception and loving-kindness were covered in this study. The interaction between differences in mindfulness practice and individual differences may reveal a more nuanced pattern and would be another area for future research to delve into.

The findings of this study have several practical implications for design and delivery of mindfulness interventions in university classrooms. The identification of three predictors, namely intrinsic motivation, conscientiousness and the observing facet of trait mindfulness, in relation to engagement and efficacy provides a starting point for tailoring such interventions to participant profiles. Targeted mindfulness intervention may show greater benefits for those higher on these predictors, while interventions for participants lower on these predictors could be adapted to support engagement more deliberately.

Specifically, for individuals lower in the observing facet of trait mindfulness, intervention design could begin with simpler awareness-based exercises and provide more guided scaffolding during early sessions to build the underlying capacity for present-moment observation. For those lower in conscientiousness, more structural support such as regular reminders, clear expectations, and brief check-ins could be implemented to compensate for lower self-directed engagement. For those lower in intrinsic motivation, interventions could draw on Self-Determination Theory to provide a clearer rationale for the practice and offer some autonomy over practice choices.

Our preliminary findings revealed the psychological profile of individuals who are more likely to be engaged in and benefit from mindfulness interventions. Overall, the significant psychosocial variables uncovered here, namely intrinsic motivation, trait mindfulness, in particular the observing facet, and conscientious personality, add to our understanding of the type of participants that might be more suited for mindfulness practice in the classroom.
